# Effectiveness of a toothpaste and a serum containing calcium silicate on protecting the enamel after interproximal reduction against demineralization

**DOI:** 10.1038/s41598-020-80844-7

**Published:** 2021-01-12

**Authors:** Ascensión Vicente, Antonio J. Ortiz-Ruiz, Belén M. González-Paz, Yolanda Martínez-Beneyto, Luis Alberto Bravo-González

**Affiliations:** 1 Unit of Orthodontics, University Dental Clinic, University of Murcia, Murcia, Spain; 2 Department of Child Integrated Dentistry, Faculty of Medicine-Dentistry, University of Murcia, Murcia, Spain; 3 Department of Preventive and Community Dentistry, Faculty of Medicine-Dentistry, University of Murcia, Avda Marqués de los Vélez. Morales Meseguer, Clínica Odontológica Universitaria 2ºplanta, 30008 Murcia, Spain

**Keywords:** Structural biology, Materials science

## Abstract

To evaluate the effectiveness of a calcium silicate/phosphate fluoridated tooth paste and a serum compared with a toothpaste containing hydroxyapatite on protecting the enamel after interproximal reduction against demineralization. 3 sets of eleven incisors were created. The teeth underwent interproximal enamel reduction (IER) of 0.5 mm. Each set was allocated to one of three groups: (1) Brushing without toothpaste (control group); (2) Vitis toothpaste + Remin Pro; (3) Regenerate toothpaste + Regenerate Serum. The agents were applied three times a day and specimens subjected to demineralization cycles for 30 days. The weight percentages of calcium (Ca) and phosphorous (P) were quantified by X-ray microfluorescence spectroscopy. Surface microhardness measurements and electron scanning microscopy (SEM) observations were made. Ca data and the Ca/P ratio were significantly higher in Group 3 than the other groups (*p* < 0.017), while P was significantly lower in Group 3 (*p* < 0.017). No significant differences were found between Groups 1 and 2 (*p* > 0.017). Group 3 showed significantly higher microhardness values (*p* < 0.05) than Group 1. No significant differences were found for other comparisons between groups (*p* < 0.05). SEM images showed less demineralization in Group 3. The application of a calcium silicate/phosphate fluoridated tooth paste (Regenerate advance) and a dual serum (Regenerate advance enamel serum) protect the enamel with interproximal reduction against demineralization. Therefore, this treatment could be used to prevent the dissolution of hydroxyapatite after IER.

## Introduction

Interproximal enamel reduction (IER), or tooth reshaping, is a procedure used in orthodontic treatment for creating extra space, especially in patients treated with clear aligners^1^.

The surface layer of the enamel is aprismatic and has higher fluoride^2^, calcium, and phosphorous content than the internal layers^3^. After IER, a less mineralized enamel surface is left exposed to the oral medium and becomes more susceptible to demineralization^4–6^. Moreover, the IER procedure roughens the surfaces, predisposing them to bacterial plaque adhesion^5^. This implies a greater predisposition to dental erosion and caries.

Both caries and erosion are the result of mineral loss but each has a different etiology. Caries is the result of the action of bacterial plaque on sugar metabolism, which produces acids that progressively eliminate minerals from the teeth. At the same time, erosion is caused by direct contact between dietary acids and the teeth. Because of increased life expectancy and the growing consumption of acidic drinks among the population, research into dental wear has become increasingly prolific and pays special attention to acid erosion^7^.

Research involving in vitro trials of the effects of protective agents against demineralization of surfaces following IER have mainly used demineralization/remineralization caries models^1,5,6,8,9^. These trials have found that applications of fluoride varnish are effective for preventing enamel calcium and phosphate loss after IER^6,8^, while resin infiltration has been shown to increase enamel microhardness^1^, topical applications of tooth mousse with casein phosphopeptide amorphous calcium phosphate (CPP-ACP) promote remineralization^5^, and tooth brushing with a zinc and hydroxyapatite carbonate toothpaste (Zn-CHA) favors the prevention of demineralization^9^. These trials were conducted using demineralization/remineralization models designed as open environments, where the interproximal tooth surfaces were directly exposed to the applications of the products and agents with free flow of the immersion solution over the surface. As far as we are aware, no in vitro studies of remineralizing products for use following IER have attempted to simulate the interproximal environment realistically.

Jones et al. ^10^, designed an interproximal mineralization model to evaluate the efficacy of a toothpaste containing calcium silicate, and sodium phosphate salts (1450 ppm fluoride as sodium monophosphate), used together with a dual gel phase system composed of calcium silicate and sodium phosphate salts in one phase and sodium fluoride in the second phase. These products are based on bioactive technologies that aim to favor tissue repair and regeneration. Calcium silicate can be transformed into hydroxyapatite deposited on both acid-eroded and sound enamel^7^. Various studies have demonstrated the effectiveness of these agents for protecting acid-softened tooth enamel^7,10–14^. Another type of agent based on bioactive technologies takes the form of toothpastes or creams containing hydroxyapatite. It has been suggested that the coating effect of micro-structured hydroxyapatite nanoparticles reintegrates the enamel with a biomimetic film, reproducing the structure and the morphology of the enamel’s biological hydroxyapatite^15^.

As none of these products with bioactive potential have been evaluated as protective agents on enamel surfaces after interproximal reduction, the aim of this in vitro study was to perform both quantitative and qualitative evaluations of the efficacy of a calcium silicate/phosphate fluoridated tooth paste (REGENERATE ADVANCE TOOTH PASTE, Enamel Science, France) and a dual serum formulation (REGENERATE ADVANCE ENAMEL SERUM, Enamel Science, France) compared with the use of a toothpaste (VITIS ANTICARIES, Dentaid, Spain) used together with a cream (REMIN PRO, VOCO, Germany) containing hydroxyapatite and fluoride on protecting the enamel after interproximal reduction against demineralization. An interproximal dental demineralization/remineralization model was used, subjecting specimens to demineralization cycles.

## Materials and methods

### Specimen preparation

The study protocol was approved by the University of Murcia (Spain) bioethics committee. The study used 33 permanent lower incisors extracted for orthodontic or periodontal reasons. All methods were carried out in accordance with relevant guidelines and regulations. An informed consent was obtained from all subjects or parents and/or legal guardian in case of under 18 years old patients.

Teeth were excluded if they presented caries, demineralization white spots, enamel defects, restorations, excessive wear, or fractures. After extraction, the teeth were placed in a 0.1% thymol solution for 24 h, after which they were stored in distilled water changed daily until the teeth were taken for use in the study. All teeth were used within 30 days of extraction.

In a rectangular plastic container, an initial set-up was made using modeling clay (Art. 70 JOVI, S.A, Barcelona, Spain) to ensure that the points of interdental contact were correct. Afterwards, acrylic was added to the plastic tray to fix the teeth in position. Three sets of eleven incisors each were created by this method.

### IER procedure

IER of the mesial and distal tooth surfaces was performed, with the exception of the distal surfaces at either end of each set. In this way, a total of 60 tooth surfaces underwent IER following the method described by Vicente et al.^6^.

The IER procedure was performed with a 0.5 mm cylindrical diamond bur (F.G 8392-016 fine grain 30 mm Komet Dental, Gebr. Brasseler GmbH & Co, Lemgo, Germany) under water refrigeration. During the procedure, the adjacent tooth surfaces were protected with a metal matrix (HAWE STEEL MATRIX BANDS, Kerr. Japan). The enamel reduction obtained was checked using a gauge measure (IPR-DISTANCE CONTROL. INTENSIV S, Montagnola, Switzerland). A 0.5 mm gauge was used when the adjacent surface was still intact and a 1 mm gauge when it had already undergone enamel reduction. A fresh bur was used after reducing each batch of 20 surfaces. The procedure was carried out by a single clinician (B.M.G).

### Experimental groups

Each set of incisors consisting of 11 incisors and 20 surfaces with IER (n = 20) were assigned to an experimental group establishing the following groups: Group 1: tooth brushing without toothpaste (Control Group); Group 2: Vitis anticaries toothpaste + Remin Pro; Group 3: Regenerate tooth paste + Regenerate Serum.

Table 1 summarizes the composition of each product evaluated.

**Table 1:** Composition of the remineralizing agents evaluated.

Agent	Composition
Vitis anticaries	0.45% Hydroxyapatite, 1.10% Sodium Monofluorophosphate (1450 ppm F-), 10% Xylitol, Aqua, Hydrated Silica, Sorbitol, Glycerin, Titanium Dioxide, Sodium Lauryl Sulfate, Xanthan Gum, PEG-40 Hydrogenated Castor Oil, Menthone Glycerin Acetal, Sodium Saccharin, Citric Acid, Sodium Methylparaben, Potassium Acesulfame, Aroma
Remin Pro	Hydroxyapatite, Sodium Fluoride (1450 ppm F-), Xylitol
Regenerate advance toothpaste	Glycerin, Calcium Silicate, PEG-8, Hydrated Silica, Trisodium Phosphate, Sodium Phosphate (1450 ppm F-), Aqua, PE-60, Sodium Lauryl Sulfate, Sodium Monofluorophospate, Aroma Flavour, Synthetic Fluorphlogopite, Sodium Saccharin, Polyacrylic Acid, Tin Oxide, Limonene, C177891
Regenerate advance enamel serum	*NR-5TM Serum*: Glycerin, Calcium Silicate, PEG-8, Trisodium Phosphate, Sodium Phosphate, Aqua, PEG-60, Sodium Lauryl Sulfate, Sodium Monofluorophosphate, Aroma, Hydrated Silica, Synthetic Fluorphlogopite, Sodium Saccharin, Polyacrylic Acid, Tin Oxide, Limonene, CI 77891
	*Activator Gel*: Aqua, Glycerin, Cellulose Gum, Sodium Fluoride (1450 ppm F), Benzyl Alcohol, Ethylhexylglycerin, Phenoxyethanol, CI 42090

### Experimental procedure

The incisor sets remained submerged in artificial saliva at 37° for the 30-days experiment period, renewing the saliva every 48 h. Three times a day the sets were removed from the saliva and placed in a demineralizing solution for 2 h. Then they were removed from the solution and without washing nor drying the buccal surfaces of the teeth were brushed manually with a medium hard toothbrush (VITIS, Dentaid, Spain) for 60 s using the modified Bass technique. Control Group teeth were brushed without toothpaste. Group 2 specimens were brushed with Vitis anticaries toothpaste, applying Remin Pro paste with the finger after each brushing, letting it act for 3 min. Group 3 teeth were brushed with Regenerate toothpaste. For the first 3 days, following the last daily brushing, Regenerate advance enamel serum was applied, using a tray, for 3 min. All procedures were carried out by the same researcher (B.M.G.), following the manufacturers’ instructions.

The composition of the artificial saliva used as storage medium was: 1% carmellose sodium, 13% sorbitol, 0.12% potassium chloride, 0.084% sodium chloride, 0.005% magnesium chloride hexahydrate, 0.015% anhydrous calcium chloride, 0.017% di-basic potassium phosphate, 0.1% and sodium nipagin. The saliva pH level was adjusted and maintained at 6.57.

The composition of the demineralizing solution was as follows: 2.2 mM calcium chloride (CaCl_2_·2H_2_O); 2.2 mM monosodium phosphate (NaH_2_PO_4_·_7_H_2_O); 0.05 mM lactic acid; the pH was adjusted to 4.5 with sodium hydroxide (NaOH) at 50%. The titratable acidity was 0.04%.

### Specimen preparation for X-ray microfluorescence analysis

The teeth were sectioned horizontally using a diamond disc (KOMET DENTAL, Gebr. Brasseler GmbH & Co. Lemgo. Germany) and a hand piece at the level of the cemento-enamel junction and along their axes, to separate the mesial and distal surfaces.

After cutting and before analysis, all surfaces were washed in distilled water and subjected to an ultrasonic bath for 30 s at room temperature.

An ORBIS Micro-XRF analyzer (EDAX, AMETEK, Berwyn, PA 19312, USA) with a solid detector of Si (Li) that was cooled with liquid nitrogen was used for the analysis of non-destructive elements. The system focuses the X-rays from a rhodium target anode with a polycapillary focusing optic. The detector has an active area of 30 mm^2^ and a 125 μm beryllium window. The acquisition system was the Orbis Vision software (version 2.0). The analysis was carried out in a vacuum of 7–8 hPa (mbar) at 40 kV and 900–1000 μA. The precision of the analyzer was 150–200 eV”.

Samples were coded so that the technician was blind.

The weight percentages of calcium (Ca) and phosphorous (P) were evaluated. Three areas of the interproximal surface were measured in each sample, subsequently the mean value was obtained for each specimen. From the Ca and P percentages, the stoichiometric Ca/P ratio was calculated applying the following formula: Ca (mol)/P (mol) % = [Ca (% weigth)/40.08 (g/mol)]/[P (% weigth)/30.97 (g/mol)], in which the atomic masses of Ca and P are 40.08 and 30.97, respectively.

### Microhardness measurement

After X-ray microfluorescence analysis, fifteen specimens in each group were used to perform surface microhardness (SMH) measurements.

SMH measurements were made with a Vickers diamond under a load of 200 g using a Microhardness Tester FM-310 (FUTURE TECH CORP., Kawasaki, Japan). The force was applied during 20 s. Each specimen was indented three times and the mean Vickers hardness value was determined for each sample.

### Specimen preparation for scanning electron microscopy (SEM)

After X-ray microfluorescence analysis, five specimens in each group were placed on plates and covered in a fine layer of gold (BIO-RAD Polaron SEM Coating System) in a vacuum chamber with a voltage of 2.5 kV and intensity of 20 mA. The covering time was 4 min per plate. The samples were examined under SEM (JSM-6100; Jeol Ltd., Tokyo, Japan) at 20 kV with × 1000 magnification. The most representative images were captured and recorded.

### Statistical analysis

Statistical analysis was performed with SPSS 19.0 software (IBM SPSS Inc., New York, USA).

The Kolmogorov–Smirnov normality test and Levene’s test of homogeneity of variance were applied to the weight percentage values for Ca and P and the stoichiometric Ca/P ratio. As data failed to fulfill the criteria for normal distribution (*p* < 0.05) or homogeneity of variance (*p* < 0.05), they were analyzed with the Kruskal–Wallis (*p* < 0.05) and Mann–Whitney tests applying Bonferroni correction (*p* < 0.017).

As SMH data fulfill the criteria for normal distribution (*p* > 0.05) and homogeneity of variance (*p* > 0.05), they were analyzed by means variance analysis (ANOVA) for one factor and the Tukey test (*p* < 0.05).

## Results

### X-ray microfluorescence analysis

The Kruskal–Wallis test identified the presence of statistically significant differences in weight percentages of Ca (*p* = 0.00), P (*p* = 0.00) and stoichiometric Ca/P ratio (*p* = 0.00) between the study groups.

The Mann–Whitney test showed that the weight percentage of Ca and stoichiometric Ca/P ratio in Group 3 (Regenerate tooth paste + Regenerate Serum) were significantly higher than in Group 2 (Vitis anticaries toothpaste + Remin Pro) (*p* = 0.000 and *p* = 0.000, respectively), and the Control Group (*p* = 0.000 and *p* = 0.000, respectively). The weight percentage of P was significantly lower in Group 3 (Regenerate tooth paste + Regenerate Serum) compared with Group 2 (Vitis anticaries toothpaste + Remin Pro) (*p* = 0.000) and the Control Group (*p* = 0.000). (Table 2).

**Table 2:** Weight percentage of Ca, P and Ca/P ratio (mol/mol).

Groups	Ca				P				Ratio Ca/P			
	Mean ± SD	Median	Range		Mean ± SD	Median	Range		Mean ± SD	Median	Range	
Control	32.54 ± 0.22	32.47	0.73	A	23.19 ± 0.15	23.33	0.5	A	1.08 ± 0.01	1.07	0.05	A
Vitis anticaries toothpaste + Remin Pro	32.65 ± 0.36	32.59	1.85	A	23.22 ± 0.22	23.25	1.12	A	1.08 ± 0.02	1.08	0.12	A
Regenerate toothpaste + Regenerate Serum	35.05 ± 1.86	35.36	4.74	B	21.81 ± 1.11	21.58	2.84	B	1.24 ± 0.12	1.23	0.33	B

In each column, capital letters indicate statistically significant differences (*p* < 0.017).

No significant differences were found between the Control Group and Group 2 (Vitis anticaries toothpaste + Remin Pro) in the weight percentage of Ca (*p* = 0.201), P (*p* = 0.369) or Ca/P ratio (*p* = 0.301). (Table 2).

### SMH measurement

The mean of SMH values are presented in Table 3. One-way ANOVA results indicated significant differences between groups (*p* = 0.03) and the Tukey test showed that Group 3 (Regenerate tooth paste + Regenerate Serum) showed significantly higher microhardness values (*p* < 0.05) than the Control Group. No significant differences were found either the Control Group and Group 2 (Vitis anticaries toothpaste + Remin Pro) (*p* = 0,27), or Group 2 (Vitis anticaries toothpaste + Remin Pro) and Group 3 (Regenerate tooth paste + Regenerate Serum) (*p* = 0.31).

**Table 3:** Vickers surface microhardness.

Groups	Mean ± SD	Median	Range	
Control	296.4 ± 67.6	332.1	167.7	A
Vitis anticaries toothpaste + Remin Pro	334.2 ± 51.5	336.2	185.5	
Regenerate toothpaste + regenerate serum	370.2 ± 55.6	352.6	149.8	B

Capital letters indicate statistically significant differences between groups (*p* < 0.05). The unmarked group did not show significant differences with any other (*p* > 0.05).

### SEM

**Figure 1:**
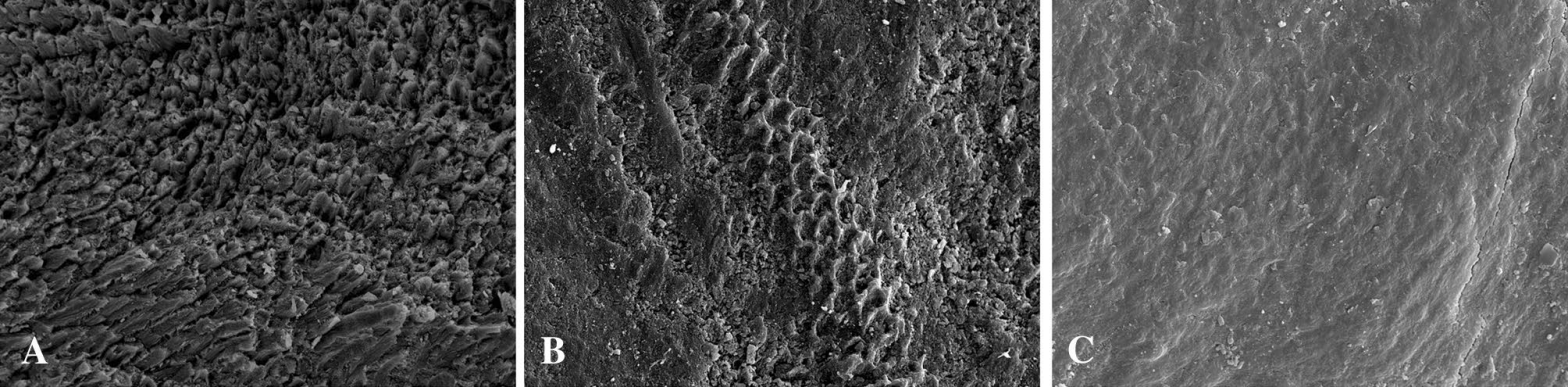
SEM (JSM-6100; Jeol Ltd., Tokyo, Japan) Images × 1000: (**A**) Control group; (**B**) Vitis anticaries toothpaste + Remin Pro; (**C**) Regenerate toothpaste + Regenerate Serum.

In the Control Group, in which teeth were brushed without any toothpaste, greater tissue dissolution was observed than in the other two groups, whereby SEM images presented a much clearer demineralization pattern (Fig. 1A). In Group 2, brushed with Vitis anticaries followed by an application of Remin Pro, SEM images reflected a slighter and more irregular process of demineralization. On the surface of the same tooth, destruction showed a preference for the center of the prisms in some areas, while in others it was observed an indiscriminate erosion or a slight demineralization pattern (Fig. 1B). The enamel brushed with the Regenerate dual system (Group 3), presented less affectation by the demineralization cycles than in the other two groups, with less tissue dissolution, whereby demineralization lesions were found to be at an earlier stage (Fig. 1C).

## Discussion

IER involves a loss of tissue and an increased risk of demineralization^4–6^. The thickness of the enamel in the interproximal area is less than on the rest of the crown, making it important to minimize mineral loss in these areas in order to protect the surfaces^10^. To date, in vitro studies evaluating the efficacy of remineralization agents for use after IER have conducted their experiments using open environmental models^1,5,6,8,9^. However, the amount of remineralization obtained with these models could be greater than observed in real in vivo situations. An interproximal model simulating the actual proximity of natural teeth will provide a more reliable reproduction of saliva flow and the behavior of oral care products in interproximal areas^14^. The active ingredients in the products and in saliva in interproximal situations interact with the enamel surface with greater difficulty than when applied directly to in vitro specimens, or when specimens are immersed in the agents allowing free flow of the solution over the surface^10^. For this reason, the present experiment applied surface treatments and demineralization cycles with the teeth mounted in acrylic blocks arranged to simulate their actual proximity in the oral cavity.

The teeth used were lower incisors, as these teeth are the most often treated with this type of procedure in clinical situations. It has been suggested that the use of incisors of different provenance, extracted from patients of different ages, could introduce some variability in the results^4^, as these factors are a source of variation in the thickness of the enamel layer and its composition. Nevertheless, IER will have a homogenizing effect because, in spite of the fact that with age the mineral density and weight percentages of Ca and P increase in the outer enamel layer, this does not occur in the same way in the middle and inner layers^3^.

Advances in the formulation of traditional fluoride toothpastes have mainly consisted of incorporating calcium or calcium salts, which can strengthen the product’s fluoride release, in turn boosting fluoride retention in the oral cavity. In addition, calcium salts can act as additional sources of calcium and reduce the demineralization process or promote enamel remineralization. In this context, based on the concept of bioactive materials for bone repair and regeneration, bioglass and especially calcium silicate type materials are increasingly used in dentistry^16^.

The aim of the present work was to evaluate the effectiveness of a toothpaste containing calcium silicate (REGENERATE ADVANCE TOOTH PASTE) together with a serum (REGENERATE ADVANCE ENAMEL SERUM), for protecting and remineralizing enamel after interproximal reduction and demineralization cycles, compared with another toothpaste (VITIS ANTICARIES) together with a cream containing hydroxyapatite (REMIN PRO).

Enamel Ca and P concentrations were determined by means of X-ray microfluorescence analysis. We have used this technique because, under ambient conditions, it is more suitable than Scanning Electron Microscopy–Energy Dispersive X-ray spectroscopy (SEM–EDX) for detecting chemical elements that have a higher atomic number than aluminum. X-ray microfluorescence analysis provides information at a depth greater than 5 μm while SEM–EDX only provides information on the surface composition. The Ca signal in the X-ray microfluorescence originates in the outermost 30 μm, therefore this technique gives more representative information on the composition of the enamel^17^. As a measure of the degree of enamel mineralization, the value of SMH was also used^13^. SEM images were used to morphologically assess the ability of the products used in our study to protect and remineralize enamel.

The results showed that the weight percentage of Ca and the Ca/P ratio in the group using Regenerate toothpaste and serum were significantly higher than with Vitis anticaries toothpaste and Remin Pro and the control group. So, the Regenerate system prevented mineral loss from enamel, a finding confirmed both in the microhardness test where the values ​​were significantly higher than in the control group and in SEM images, where the surface was seen to be less affected by the demineralization cycles, with less tissue dissolution than in the other groups.

Previous SEM studies have suggested that IER produces a rougher enamel surface often presenting furrows, grooves and scratches^1,6,8^. It has been shown that calcium silicate particles have a particular affinity for high energy sites, such as scratches and other surface defects. For this reason, the enamel surface produced by interproximal reduction is an ideal substrate for applying this type of material. It is thought that the calcium ions in calcium silicate are exchanged with H^+^ in the surrounding fluid, which leads to silanol (Si–OH) formation in the surface layer. This causes an increase in pH and eventually the formation of a negatively charged surface with the Si–O functional group. For this reason, the solution in the immediate vicinity of the surface must therefore be depleted of protons and enriched with calcium. When combined with phosphate ions from surrounding saliva, hydroxyapatite precipitation onto the surface of the calcium silicate takes place. Therefore, the protection and remineralization mechanism is due to a combination of calcium release, pH buffering, and hydroxyapatite formation^12^. The nucleated hydroxyapatite acts as a sacrificial layer to the underlying enamel^13^.

The present study’s results cannot be compared with other works involving IER because there are no studies in which these products have been applied after this procedure, but can be compared with others investigating the use of the products applied to intact and/or demineralized enamel. Wood et al., using an in vitro interproximal model, found greater demineralization prevention with the combined application of calcium silicate-based paste and serum compared with a conventional fluoride toothpaste (1450 ppm F-), and with a non-fluoride toothpaste^14^. Hornby et al. found that serum application boosted the efficacy of toothpaste in just a single day^13^. Other studies, using both an in vitro interproximal model^10^ and an in situ/ex vivo model^11^, have also reported the greater effectiveness of these products compared with a conventional fluoride toothpaste for re-hardening enamel previously exposed to acid attack. It would appear that acid-eroded enamel surfaces favor calcium silicate deposits to a greater extent than sound surfaces, which in turn favors the reparative potential of surfaces affected by erosion^7^. Ionta el al. in an in situ/ex vivo study, found that calcium silicate toothpaste reduced enamel loss provoked by an acid challenge, but not by an acid and brushing challenge. The authors suggested that the 5-day duration of the study was perhaps insufficient to allow the formation of a hydroxyapatite layer capable of resisting the abrasive action of tooth brushing^18^.

Regarding phosphorous (P) loss observed in the group treated with Regenerate tooth paste and serum, it could be that this element was consumed through the formation of some compound that was not incorporated into the tissue in a stable way, which was then eliminated by the brushing action, although further research is needed to confirm this.

In SEM images of control group specimens, brushed without paste, greater tissue dissolution was observed than in the other groups, presenting more evident demineralization. As well as greater demineralization, these images showed greater exposure of the enamel prisms than observed by the present research team in an earlier work, in which specimens were not subjected to brushing^6^. It is likely that the abrasion provoked by brushing acid-softened enamel eliminates part of the demineralized enamel^19^.

Although images of the group that received Remin Pro after brushing with Vitis anticaries toothpaste presented less demineralization than the control group, no significant differences were detected either in weight percentages of Ca, P and in the Ca/P ratio or in the microhardness values between these groups. The SMH values of the teeth treated with Vitis anticaries and Remin Pro were in an intermediate position between the SMH in the control group and in the group in which the Regenerate system was applied, since it did not show significant differences with any of them. As far as we are aware, no other research has investigated the application of these products to enamel following interproximal reduction. Nevertheless, various authors concur that hydroxyapatite-based toothpastes show similar efficacy to fluoride toothpastes^20,21^. As for Remin Pro, its use has been evaluated both as a prevention and treatment for demineralization lesions. Tahmasbi et al. conducted an in vitro trial, in which teeth underwent demineralization cycles for 14 days, finding no significant differences in enamel microhardness between teeth treated with Remin Pro and a control group^22^. Meanwhile, patients with post-orthodontic white spot lesions treated with Remin Pro, achieved a significant decrease in white spot area and a significant increase in mineral content^23,24^. Moreover, Esfahani et al. found that in vitro applications of Remin Pro significantly increased microhardness of previously demineralized enamel^25^. Remin Pro contains calcium and phosphate in the form of hydroxyapatite with a capacity to fill enamel surface lesions^24^, and it may be that its application is more effective on previously demineralized enamel than as a measure against mineral loss.

Based on our results, although it is an in vitro study, we can affirm that the dentists when performing an IER procedure must be aware that are exposing an enamel surface more susceptible to demineralization and therefore its protection to the oral environment is important. In this work the dual system Regenerate was effective in protecting the enamel with interproximal reduction against demineralization.

## Conclusions

Although in vivo trials are needed to confirm the results, the present findings suggest that the application of a calcium silicate/phosphate fluoridated tooth paste (REGENERATE ADVANCE) and a dual serum (REGENERATE ADVANCE ENAMEL SERUM) protect the enamel with interproximal reduction against demineralization. Therefore, this treatment could be used to prevent the dissolution of hydroxyapatite after IER.
